# Biochemical analysis of packing and assembling heptad repeat motifs in the coronavirus spike protein trimer

**DOI:** 10.1128/mbio.01203-24

**Published:** 2024-10-23

**Authors:** Jun Kobayashi, Kazuhiko Kanou, Hiyori Okura, Tahmina MST Akter, Shuetsu Fukushi, Shutoku Matsuyama

**Affiliations:** 1 Research Center for Influenza and Respiratory Viruses, National Institute of Infectious Diseases, Tokyo, Japan; 2 Department of Quality Assurance, Radiation Safety, and Information Management, National Institute of Infectious Diseases, Tokyo, Japan; 3 Department of Virology I, National Institute of Infectious Diseases, Tokyo, Japan; University of Pennsylvania, Philadelphia, Pennsylvania, USA; Albert Einstein College of Medicine, Bronx, New York, USA

**Keywords:** coronavius, spike, fusion, protease, CEACAM1a, ACE2, SARS-CoV-2, MHV, intermediate

## Abstract

During a coronavirus infection, the spike protein undergoes sequential structural transitions triggered by its receptor and the host protease at the interface between the virus and cell membranes, thereby mediating membrane fusion. After receptor binding, the heptad repeat motif (HR1/HR2) within the viral spike protein bridges the viral and cellular membranes; however, the intermediate conformation adopted by the spike protein when drawing the viral and cellular membranes into close proximity remains unclear due to its transient and unstable nature. Here, we experimentally induced conformational changes in the spike protein of a murine coronavirus by incubating the virus with its receptor, followed by exposure to trypsin. We then treated the virus/receptor complex with proteinase K to probe the tightly packed core structure of the spike protein. The conformations of the spike protein were predicted from the sizes of the protease digestion products detected by western blot analysis. Upon receptor binding, two bands (each showing different reactivity with a fusion-inhibiting HR2-peptide) were detected; we propose that these bands correspond to the packed and unpacked HR1/HR2 motifs. After trypsin-mediated triggering, measurement of temperature and time dependency revealed that packing of the remaining unpacked HR1/HR2 motifs and assembly of three HR1 motifs in a trimer occur almost simultaneously. Thus, the trimeric spike protein adopts an asymmetric-unassembled conformation after receptor binding, followed by direct assembly into the post-fusion form triggered by the host protease. This biochemical study provides mechanistic insight into the previously unknown intermediate structure of the viral fusion protein.

**IMPORTANCE:**

During infection by an enveloped virus, receptor binding triggers fusion between the cellular membrane and the virus envelope, enabling delivery of the viral genome to the cytoplasm. The viral spike protein mediates membrane fusion; however the molecular mechanism underlying this process is unclear. This is because using structural biology methods to track the transient conformational changes induced in the unstable spike trimer is challenging. Here, we harnessed the ability of protease enzymes to recognize subtle differences on protein surfaces, allowing us to detect structural differences in the spike protein before and after conformational changes. Differences in the size of the degradation products were analyzed by western blot analysis. The proposed model explaining the conformational changes presented herein is a plausible candidate that provides valuable insight into unanswered questions in the field of virology.

## INTRODUCTION

The coronavirus spike (S) protein mediates attachment of the virus to host cells, followed by fusion of the viral and cellular membranes prior to insertion of the viral genome into the host cell cytoplasm (1). The S protein is a class I fusion protein that contains two subunits: S1 and S2 (2). S1 binds to the receptor on the cell, whereas S2 mediates fusion of the viral and cellular membranes (3). Coronaviruses such as severe acute respiratory syndrome coronavirus (SARS-CoV), human coronavirus 229E (HCoV-229E), and mouse hepatitis virus type 2 (MHV-2) possess an uncleaved 180 kDa S protein (4–6), whereas severe acute respiratory syndrome coronavirus-2 (SARS-CoV-2) and MHV-A59 possess an S protein that is cleaved at the S1/S2 site by a cellular protease (called furin) during biogenesis (7, 8).

Virological and biochemical analyses revealed that activation of the S protein requires a two-step conformational change induced by receptor and protease (9, 10). Receptor binding destabilizes the interface between S1 and S2, leading to unmasking of the fusion peptide (FP) in the S2 subunit (11). Structural analyses, including cryo-electron microscopy (cryoEM) and X-ray crystallography, have identified the structure of the S protein in its pre-fusion, receptor binding, and post-fusion states (12–20). The molecular rearrangements that occur during the early stages of the receptor-binding step have been well-characterized by high-resolution cryoEM (18, 19). Although the stable regions of S1 and S2 have been well-characterized, the stalk region of S2 remains largely uncharacterized due to its disordered structure. A study using molecular dynamics simulation and cryoEM tomography revealed that the stalk region of the SARS-CoV-2 S2 subunit in the pre-fusion state contains three flexible hinges (21). Our previous studies using MHV-2, a liposome flotation assay (10), and electron microscopy (22) showed membrane binding of the virus in the presence of the receptor; these results suggest that the S2 subunit can expose the FP even when covered by the S1 subunit. The subsequent step is driven by proteases present at the cell surface or within the endosome; these include TMPRSS2, trypsin, elastase, and cathepsin L, all of which cleave the S protein at an S2' site or nearby (4, 7, 9, 23–25). During this step, the S2 subunit folds back on itself to form a hairpin structure, drawing the viral and cellular membranes into close proximity and resulting in lipid mixing (3, 23).

The post-fusion form of the S2 subunit adopts a central N-terminal trimeric α-helical coiled-coil structure (a trimer of heptad repeat 1, HR1) surrounded by three C-terminal helices (HR2), thereby forming a six-helix bundle (6HB) (26–28). However, the mechanism by which the HR1/HR2 regions of the trimer undergo the conformational changes required to form the 6HB are not fully understood. A previous study that used cryoEM tomography to capture the intermediate form of SARS-CoV-2 S protein by preventing its refolding with a fusion-inhibiting HR2-peptide (29) showed that this intermediate seems to adopt a typical homotrimeric pre-hairpin structure comprising a three-helical bundle of HR1, without packing of HR2 between viral and cellular membranes; this is a known model for class I viral fusion proteins (3, 30). Our previous study based on biochemical observations proposed an alternative model; the asymmetric S protein trimer formed after receptor-binding contains the prepacked and unpacked HR1/HR2 motifs, within which the HR1 motifs are not assembled at a center; then, the host protease cleaves the S2′ site, which triggers the second conformational change to facilitate direct assembly of the prepacked and unpacked HR1/HR2 motifs, followed by the formation of 6HB (22). Here, we provide additional data to support our previous model in an attempt to explain how the spike protein undergoes the conformational changes that drive membrane fusion.

## RESULTS

### Conformational changes in the S protein at each step of the activation process

**Fig 1:**
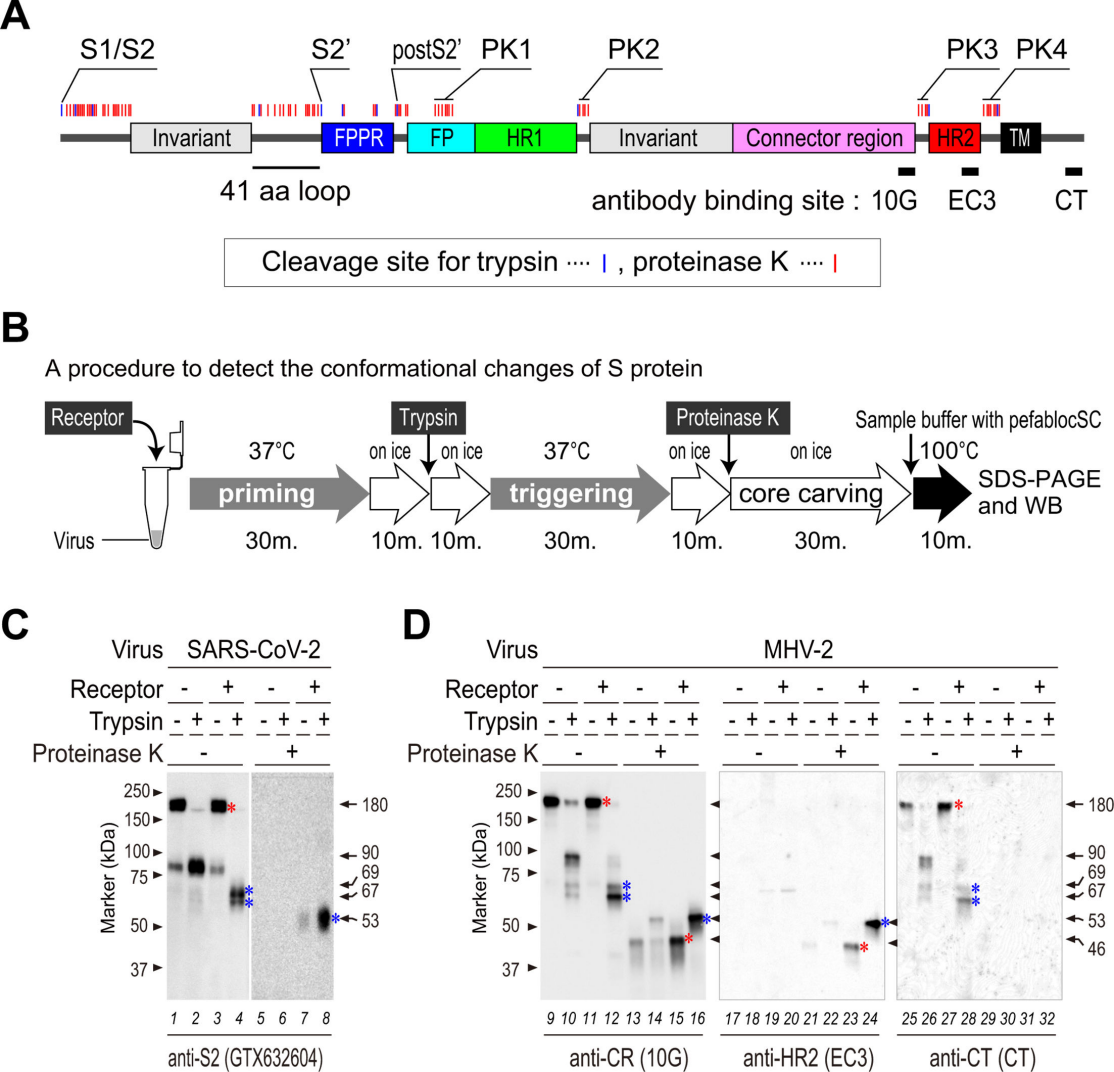
*In vitro* activation of the SARS-CoV-2 and MHV-2 spike (**S**) proteins. (**A**) Schematic diagram showing organization of the MHV-2 S2 subunit. The configuration of domains is based on data from the recent SARS-CoV-2 study (20). FPPR, fusion peptide proximal region; FP, fusion peptide; HR1/HR2, heptad repeats; CR, connector region; TM, transmembrane domain. The three trypsin cleavage sites (S1/S2, S2', and postS2'), the four predicted proteinase K cleavage sites (PK1–PK4), and the three linear epitopes recognized by antibodies 10G, EC3, and CT are indicated. (**B**) Experimental flow chart showing induction of conformational changes in the viral S protein. (**C**) Conformational changes in the SARS-CoV-2 S protein. Virus was incubated at 37°C for 30 min with 50 µg/mL soluble hACE2 (receptor), followed by 10 µg/mL trypsin at 37°C for 30 min. To carve out the tightly packed S protein core, reaction mixtures were treated with 100 µg/mL proteinase K on ice for 30 min. Samples were then subjected to SDS-PAGE (10% gels), followed by western blot analysis with an antibody specific for the SARS-CoV-2 S2 subunit. (**D**) Conformational changes in the MHV-2 S protein. Similar to panel B, virus was treated with 50 µg/mL soluble mCEACAM1a (receptor), followed by digestion by trypsin and proteinase K. SDS-PAGE and western blot analysis were performed using the indicated antibodies. (**C and D**) The bands detected after conformational changes primed by the receptor (i.e., 180, 69, and 46 kDa) are marked by red asterisks, and those related to the post-fusion state triggered by trypsin (i.e., 69, 67, and 53 kDa) are marked by blue asterisks. These asterisks appear in all of the western blot panels presented in this study.

As in our previous study (22), most of the experiments described herein were conducted using MHV-2 because it possesses an uncleaved S protein (10) that does not undergo conformational changes in the absence of protease after receptor binding; this makes it suitable for analysis of intermediate conformations. The size of the protease digestion products at each of the activation steps was analyzed by western blotting because the protease cleavage site in the S protein shifts in response to conformational changes; i.e., the ability of proteases to recognize subtle differences on the S protein surface makes them useful tools for exploring S protein conformations. In particular, previous studies show that proteinase K recognizes a broad spectrum of amino acid substrates, and that it cleaves the loop structures of the protein surface but not the helical structures, buried domains, or the inside of the envelope (31–34). When we predicted proteinase K-cleavable sites within the S protein in its receptor-binding state (protein data bank: 6VSJ), the surface loops were considered to be cleavable; the putative cleavage sites are depicted on the schematic diagram of the MHV-2 S protein shown in Fig. 1A The epitopes recognized by the antibodies used in this study are also depicted (Fig. 1A).

To characterize the differences in the size of protease digestion products derived from the S protein corresponding to each of the conformational changes, we incubated the virus with its receptor, followed by exposure to trypsin. The experimental flowchart is presented in Fig. 1B. The results indicate that both SARS-CoV-2 and MHV-2 possess an uncleaved S protein of 180 kDa (Fig. 1C, lane 1 and 1D, lane 9). The additional 90 kDa band observed for SARS-CoV-2 (lanes 1 and 3) was not observed for MHV-2 (lanes 9 and 11); this is because the S protein of MHV-2 is not cleaved by furin; therefore, the movement of the FP of MHV-2 is potentially restricted in the absence of protease because it is coupled to an invariant motif via a 41 amino acid loop and a fusion peptide proximal region (FPPR) (Fig. 1A). Cleavage of the 180 kDa S protein (Fig. 1C, lane 1 and Fig. 1D, lane 9) by trypsin generated a 90 kDa band (lanes 2 and 10) in the absence of the receptor but generated two different S2 fragments (67 and 69 kDa) in the presence of the receptor (lanes 4 and 12); this explains the conformational change in the S protein induced by the receptor. Subsequent proteinase K treatment of virus samples pretreated with both receptor and trypsin generated a 53 kDa band (lanes 8 and 16), which was thought to include the post-fusion 6HB structure (10). Interestingly, incubation of the MHV-2 S protein with receptor alone (i.e., without trypsin treatment) generated a proteinase K-resistant band of 46 kDa (lane 15) derived from the 180 kDa S protein (lane 11), which is involved in the receptor-primed intermediate. Although the S proteins of MHV-2 and SARS-CoV-2 undergo similar conformational changes, the unique proteinase K-resistant 46 kDa band observed for MHV-2 (lane 15) was not observed for SARS-CoV-2 (lane 7), presumably due to differences in the antibody recognition sites.

To estimate the motifs within the 46 and 53 kDa fragments, we used anti-peptide antibodies specific for the HR2 motif (EC3) and cytoplasmic tail (CT). The anti-HR2 antibody (EC3) detected only proteinase K-resistant bands (lanes 23 and 24). This may be because the epitope buried in the globule is exposed by proteinase K digestion. These bands (46 and 53 kDa) suggest that the HR2 motif resides within the stable core during the receptor binding and post-fusion states. The anti-CT antibody did not detect S proteins treated with proteinase K (lanes 31 and 32), indicating that proteinase K degrades the C-terminus of the S protein, presumably at the membrane proximal region (Fig. 1A; PK4), as described in the next paragraph.

### Deglycosylation of the proteinase K-resistant S2 core

**Fig 2:**
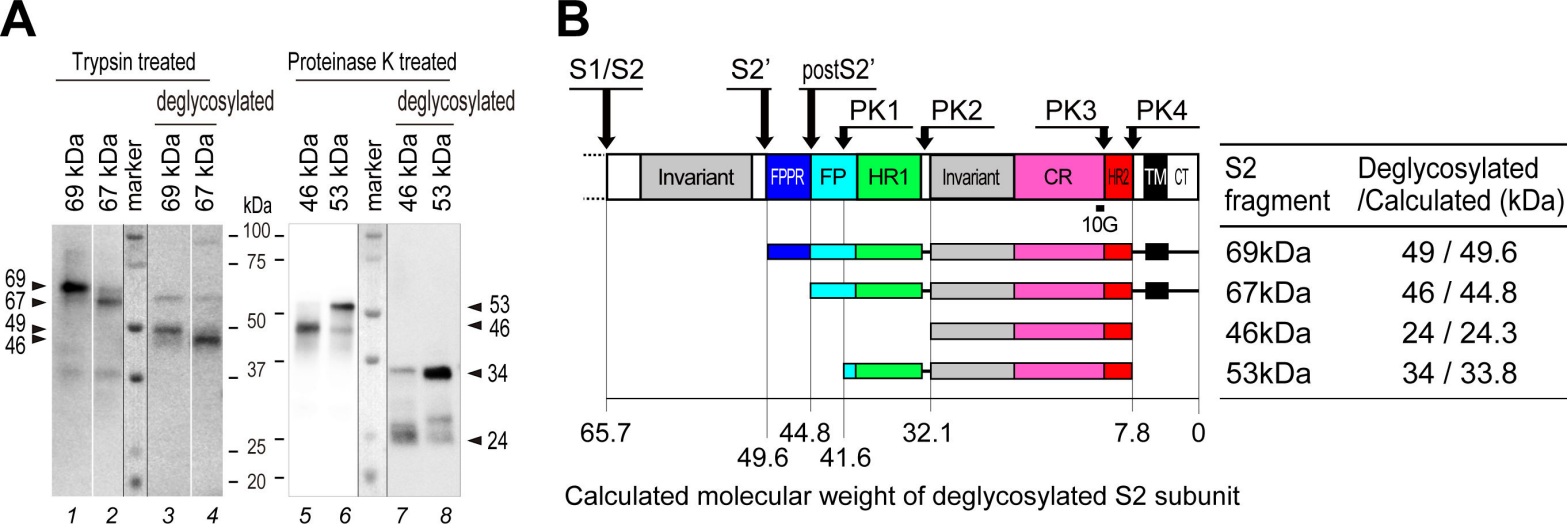
Deglycosylation of the protease digestion products derived from the MHV-2 S2 subunit. (**A**) MHV-2 samples treated with receptor, trypsin, and proteinase K (as described in Fig. 1D) were then treated with a commercial deglycosylation mix. SDS-PAGE and western blot analysis were carried out using an anti-CR antibody (MAb10G). (**B**) Schematic diagram showing the deglycosylated S protein. The size of the deglycosylated S2 subunits observed in panel A was compared with the size calculated from the amino acid sequences obtained after cleavage at the predicted sites. The epitope recognized by antibodies (MAb10G) is indicated.

To identify the protease cleavage site in the S protein, we attempted to ascertain the exact size of the deglycosylated protein rather than that of the amino-terminal sequence; this is due to the small amounts of these fragments, as well as the high concentration of proteinase including which makes it difficult to purify a sufficient amount of material from the virus solution for analysis by mass spectrometry; in addition, there was a lot of cellular protein in the mixture. Deglycosylation of the S2 fragment was carried out using a commercial kit containing five enzymes that completely remove N- and O-linked glycans from glycoproteins. MAb10G, which recognizes the connector region within S2 (Fig. 1A), was used for western blot analysis. After deglycosylation, the trypsin-generated 69 and 67 kDa products (Fig. 2A, lanes 1 and 2) shifted to 49 and 46 kDa (lanes 3 and 4), respectively, and the proteinase K-generated fragments at 46 and 53 kDa (lanes 5 and 6) shifted to 24 and 32 kDa (lanes 7 and 8), respectively. When the calculated molecular weight of the S2 fragment based on the amino acid sequence was compared with the experimentally obtained size (Fig. 2B), the result suggested that the S2 subunit forms a proteinase K-resistant core containing an invariant motif, a connector region, and a HR2 motif, but not a CT (cleaved at PK4 in Fig. 2B) after the receptor-binding step. The difference between the receptor-primed state (24 kDa) and the post-fusion state (34 kDa) is due to the degradation of the HR1 motif (Fig. 2B; PK1 and PK2); therefore, the HR1 motifs are flexible and unassembled at the center during the receptor-binding step.

### Interaction of the HR2-peptide with the S protein

To characterize the packed or unpacked status of the HR1/HR2 motif within the S2 subunit, we used fusion-inhibiting HR2-peptides, i.e., HR2-mimicking peptides (HR2-peptides): 39 amino acids of MHV (35) and 36 amino acids of SARS-CoV-2 (36). These amino acid sequences, which are derived from the HR2 motif of the S2 subunit, are known to interfere with packing of the HR1/HR2 motif, thereby inhibiting membrane fusion.

First, to confirm that the HR2-peptide suppresses virus entry into cells, we conducted a virus entry assay by measuring the amount of newly synthesized viral mRNA in cells using real-time PCR. A calibration curve showing the relationship between the number of virus infectious units and viral RNA levels in cells at 5 h post-infection was used to estimate the amount of infected virus (Fig. S1A). Of note, a previous study shows that coronaviruses enter cells via two routes: the endosomal pathway and the cell surface pathway (37). The HR2 peptide effectively inhibits SARS-CoV entry at the cell surface, but less effectively at the endosome; this is because it is likely degraded by endosomal cathepsins (38). Therefore, we needed to estimate the functionality of the HR2-peptide in the presence of an inhibitor of the endosomal pathway. To do this, we used the cysteine protease inhibitor E64d. Unexpectedly, we found that the peptide (20 µM) suppressed MHV entry into DBT cells significantly in the absence of E64d; however, and as expected, the peptide inhibited trypsin-driven viral cell entry more efficiently in the presence of E64d (Fig. S1B). Furthermore, the HR2-peptide suppressed MHV-2 cell entry in a concentration-dependent manner in the presence of E64d (Fig. S1C).

**Fig 3:**
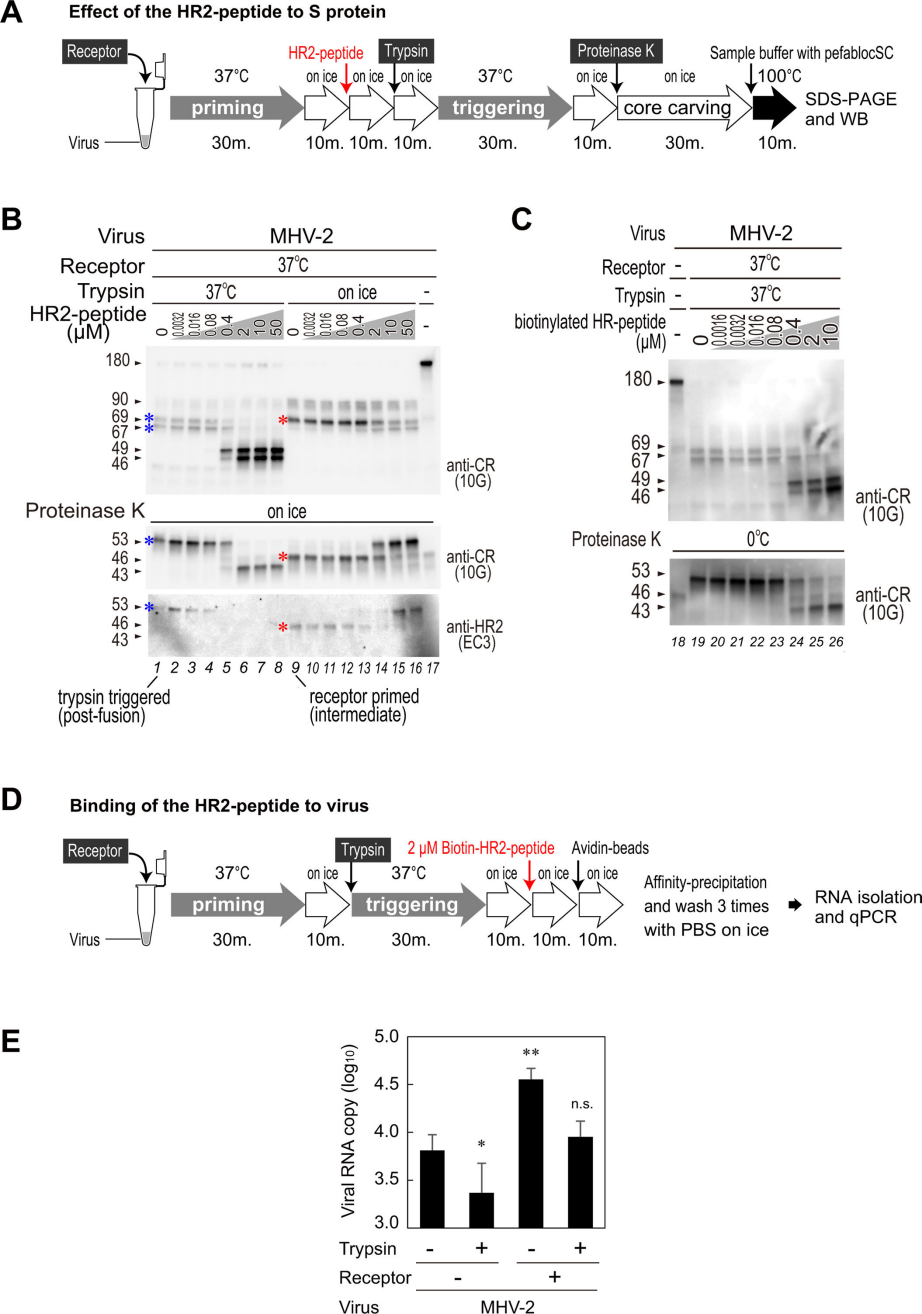
Interaction of the HR2-peptide with MHV-2 S protein. (**A**) Experimental flow chart showing treatment with the HR-2 peptide. (**B**) Effect of HR2-peptide on the conformation of the S2 subunit. During experimental activation of the S protein, the HR2-peptide (serially diluted fivefold) was added after the receptor-binding step, followed by trypsin treatment. Next, samples were treated with proteinase K to carve out the core structure. Western blot analysis was carried out using an anti-CR antibody (MAb10G). (**C**) Reactivity of the biotinylated-HR2-peptide. Biotinylated-HR2-peptide specific for MHV-2 was added to the virus after the receptor-binding step to estimate the optimum concentration of the peptide. The virus was then treated with proteinase K to carve out the core structure, followed by western blot analysis with an anti-CR antibody (MAb10G). (**D**) The experimental flow chart showing the procedure used to estimate binding of the biotinylated-HR2-peptide to the virus. (**E**) HR2-peptide binding to the virus. The reaction mixture obtained at each step of the *in vitro* conformational change experiment was mixed with 2 µM biotinylated-HR2-peptide and precipitated with streptavidin beads. Viral RNA was quantified by real-time PCR. Two-tailed Student’s *t*-tests were used to analyze the significance of differences between virus-treated and non- treated samples.

Next, we used western blot analysis to assess the effect of the HR2-peptide on S protein conformation. MHV-2 was pretreated with receptor and then exposed to various concentrations of the HR2-peptide A flow chart illustrating the experimental procedure is presented in Fig. 3A. To analyze the S protein digestion products at the receptor-binding step, trypsin treatment was carried out on ice to arrest subsequent conformational changes (Fig. 3B, lanes 9–16). By contrast, to analyze the S protein in the post-fusion state, trypsin treatment was carried out at 37°C to initiate subsequent conformational changes (Fig. 3B, lanes 1–8). After the receptor-binding step in the absence of the HR2-peptide, a 69 kDa band appeared after exposure to trypsin on ice for 10 min (Fig. 3B, upper panel, lane 9), and a 67 kDa band appeared after shifting the temperature to 37°C (Fig. 3B, upper panel, lane 1). The results support those presented in Fig. 1D. In the presence of the HR2-peptide, two bands (69 and 67 kDa) observed after trypsin-mediated cleavage on ice (Fig. 3B, upper panel, lanes 14–16) shifted to 46 and 49 kDa, respectively, at 37°C (Fig. 3B, upper panel, lanes 6–8), indicating the HR2-peptide affects the conformation of S2, resulting in the exposure of the buried cleavage site. The addition of proteinase K yielded a 43 kDa band derived from the 46 and 49 kDa bands (Fig. 3B, middle panel, lanes 6–8), which was not detected by the anti-HR2 antibody (Fig. 3B, bottom panel, lanes 6–8). This suggests that the HR2 motif is degraded by proteinase K in the presence of the HR2-peptide; thus, a cleavage site is presumed to reside at “PK3” (see Fig. 1A and 2B).

For SARS-CoV-2, an assay was also performed to confirm that the HR2-peptide suppresses virus entry into cells. The calibration curve (Fig. S2A) was used to estimate virus entry based on the amount of cellular RNA isolated from VeroE6-TMPRSS2 cells at 5 h post-infection. The HR2-peptide effectively suppressed viral entry; furthermore, the inhibitory effect was greater in the presence of E64d (Fig. S2B) and was concentration-dependent (Fig. S2C). The effect of the HR2-peptide on SARS-CoV-2 S protein was then examined by western blotting. A flow chart illustrating the experimental procedure is presented in Fig. S2D. We observed two S2 fragments at 69 and 67 kDa following trypsin-mediated cleavage; these bands were detected in the presence or absence of the HR2-peptide (Fig. S2E lanes 1–8). Subsequent treatment with proteinase K yielded a band at 53 kDa in the presence of a low concentration of the HR-peptide (lanes 1–6 and 9–14). This 53 kDa band was degraded further in the presence of 10 or 50 µM HR2-peptide, regardless of the incubation temperature (lanes 7, 8, 15, and 16), suggesting that the HR2-peptide has an effect on the SARS-CoV-2 S2 subunit; however, the unique bands observed on MHV-2 (i.e., those at 46 and 49 kDa in Fig. 3B, upper panel, lanes 6–8) were not generated.

In addition, we confirmed direct binding of HR2-peptide to the MHV-2 at each activation step by conducting an affinity-precipitation assay. First, we prepared a biotinylated-HR2-peptide and confirmed that it reacted with S2 in a manner similar to that of the non-labeled HR2-peptide (Fig. 3C). Next, biotinylated-HR2-peptide (2 µM) was added to MHV-2 after induction of S protein conformational changes, followed by precipitation using streptavidin beads (see the experimental flow chart in Fig. 3D). RNA isolated from the precipitated viruses was quantified by real-time PCR. As expected, viruses primed with receptor were precipitated by the biotinylated HR2-peptide to a greater extent than viruses obtained during the other steps (Fig. 3E).

**Fig 4:**
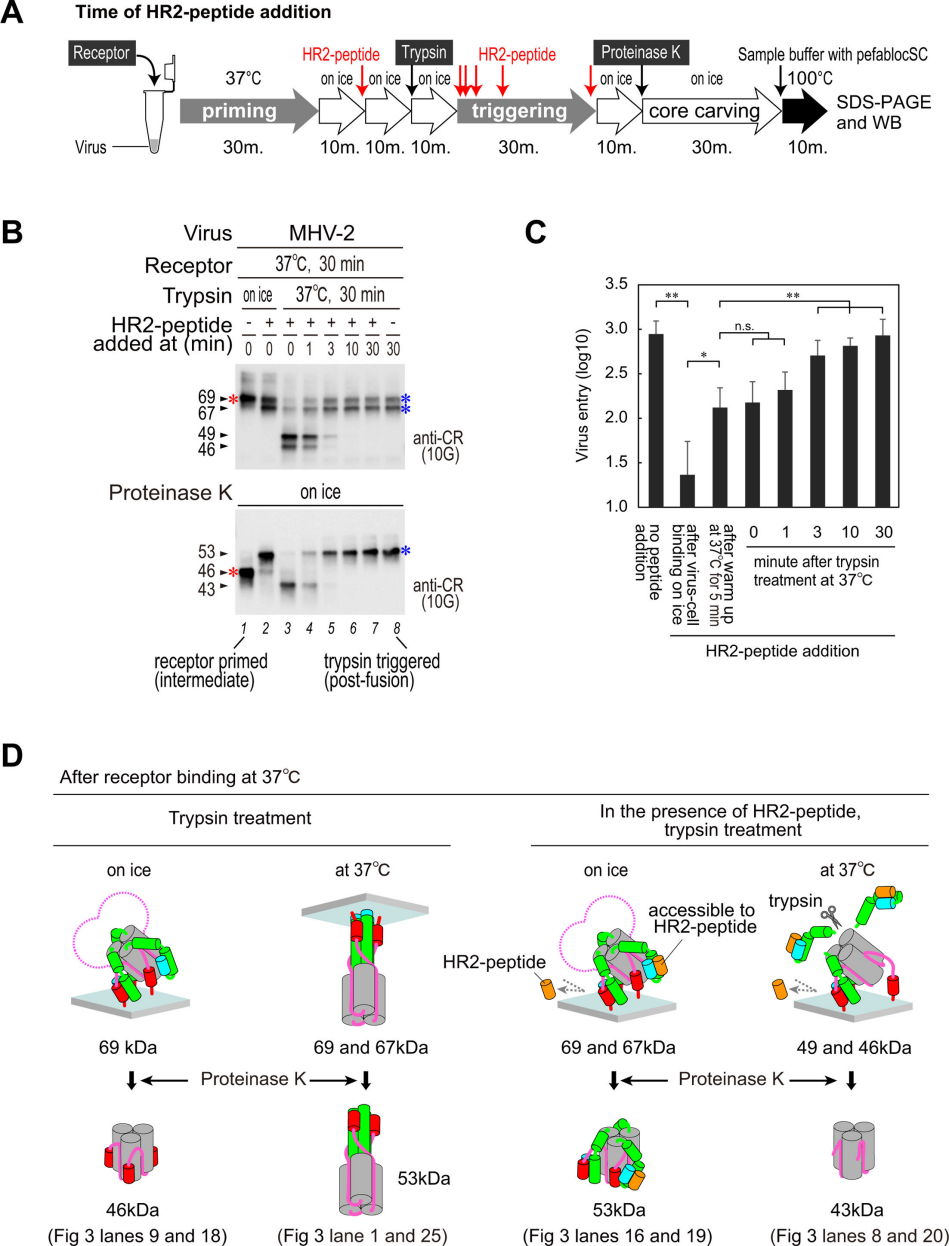
Time of HR2-peptide addition. (**A**) Experimental flow chart for the time of HR-2 peptide addition experiments. (**B**) Time of HR2-peptide addition. After the receptor-binding step, the HR2-peptide was added at the indicated times during trypsin treatment at 37°C. Western blot analysis was carried out using the MAb10G. (**C**) Inhibition of viral cell entry using HR-2 peptide. DBT cells with attached MHV-2 were treated with trypsin and incubated at 37°C to drive virus cell entry. HR2-peptide was added (final concentration, 20 µM) at the indicated times. After 5 h, cellular RNA was extracted, and newly synthesized viral RNA was quantified using real-time PCR. (**D**) Model of the S2 subunit before and after treatment with proteinase K. These models were predicted from the results shown in Fig. 3 and 4.

The time of HR2-peptide addition experiment, as shown in the experimental flow chart in Fig. 4A, again revealed that the proteinase K-resistant core of MHV-2 S protein was detected as a 46 kDa band after receptor binding (Fig. 4B, lane 1), which then shifted to a 53 kDa band after the addition of the HR2-peptide on ice (lane 2). This was further degraded to a 43 kDa protein after incubation with trypsin at 37°C (lane 3). Upon incubation with trypsin for 3 min at 37°C, the interaction between the HR2-peptide and the S protein was no longer detectable (i.e., the 43 kDa band was not detected); instead, a 53 kDa band representing the final conformation (i.e., 6HB) was detected (lane 5), suggesting that the HR1/HR2 motif was packed.

Furthermore, to clarify the step at which the HR2-peptide blocks viral entry into cells, we performed the time of HR2-peptide addition experiment using cultured cells. DBT cells were detached from the culture bottle using a non-enzymatic cell dissociation solution and then incubated with MHV-2 on ice for 30 min. The reaction performed to detect virus activation on cells was conducted in a micro-tube. HR2-peptide was added at each step of viral S protein activation to block virus-cell entry. Then, the virus-cell mixture was added to the culture medium and plated in the 96-well plate. After 5 h, cellular RNA was extracted and real-time PCR was performed to quantify the replicated viral RNA. The addition of the HR2-peptide efficiently blocked viral cell entry after the receptor-binding step on ice. Subsequent incubation of cells at 37°C for 5 min partially reduced the inhibitory effect of the HR2-peptide (Fig. 4C), indicating the HR1/HR2 motifs were partially packed. After addition of trypsin, the HR2-peptide still had ability to block the viral cell entry for a few min at 37°C, but not after incubation for 30 min (Fig. 4C), indicating the HR1/HR2 motifs were completely packed. These results suggest that HR1/HR2 motifs undergo two rounds of packing, i.e., after receptor priming and after trypsin triggering.

Models of the proteinase K-resistant core derived from the MHV-2 data are shown in Fig. 4D. After binding to the receptor, the trimer forms a stable core comprising three S2 subunits excluding the HR1 motif, as detected at 46 kDa. Upon shifting to 37°C, the three HR1 motifs assembled to form a 6HB structure (detected at 53 kDa). In the presence of the HR2-peptide, the HR1 motifs were stabilized on the core without assembling and were detected as a band at 53 kDa. Upon shifting to 37°C, the HR1 motifs are flexible and are degraded by proteinase K (detected as a band at 43 kDa).

### Prediction of the FP–HR1/HR2 complex in the intermediate state

**Fig 5:**
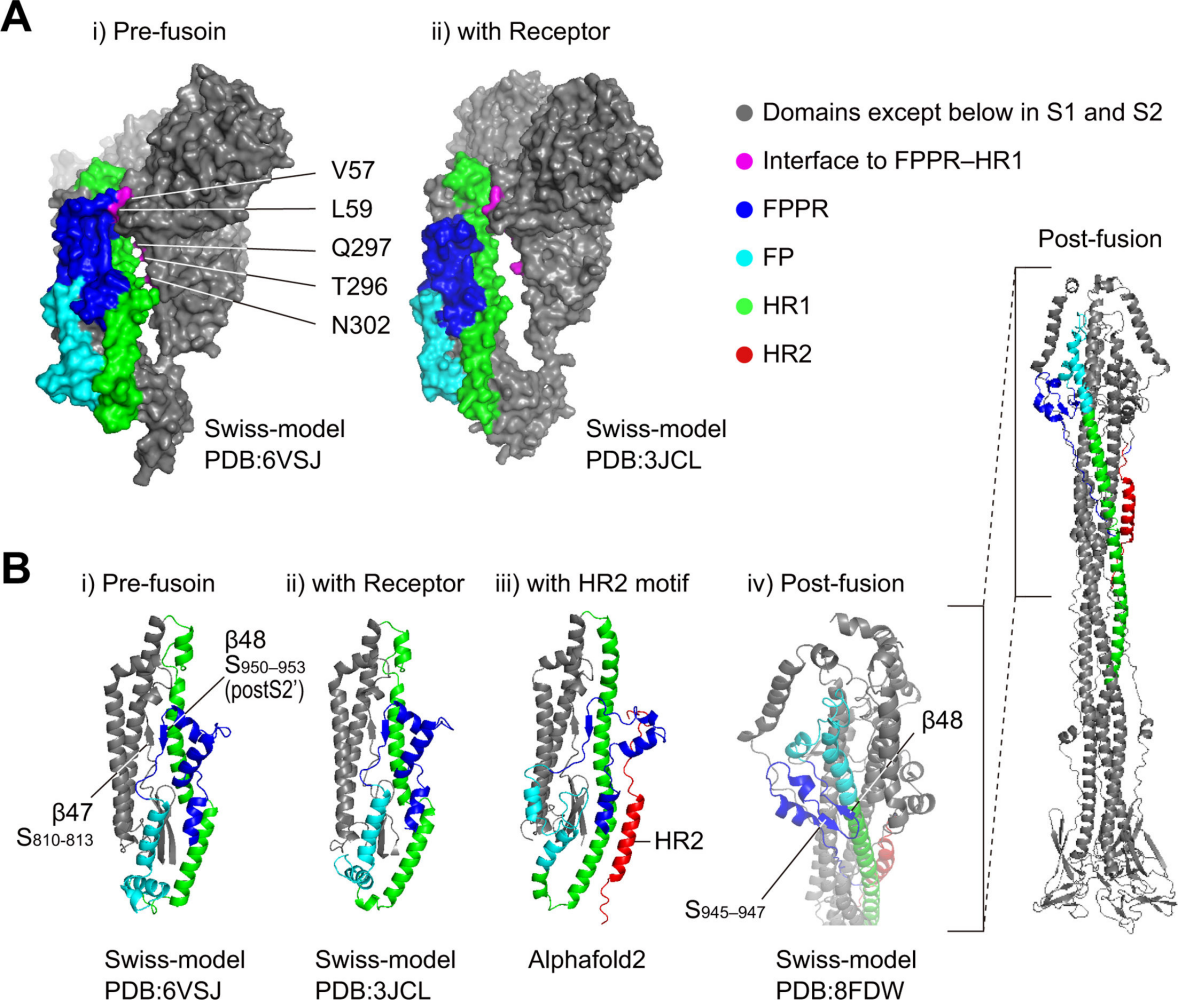
Prediction of the FPPR–HR1/HR2 complex. (**A**) The “closed” and “open” structures of the S2 subunit. The structure of the MHV-2 S protein in the pre-fusion state (**i**) and in the receptor-binding state (ii) was predicted by homology modeling (Swiss-model) based on the MHV-A59 S protein structure revealed by cryoEM. Interface residues between the FPPR–HR1 motif and other regions of the S protein in the pre-fusion state (**i**) were compared with those in the receptor-binding state (ii), as predicted by PyMol script InterfaceResidues. (**B**) The predicted FPPR–HR1/HR2 structure. From the two models in panel A, the domains around the FPPR–HR1 region were cropped and presented in (**i**) and (ii). The intermediate structure of the FPPR–HR1 region complexed with HR2 motif in the receptor-binding state was predicted using AlphaFold2 software (iii). The post-fusion state was predicted by homology modeling (Swiss-model) based on the SARS-CoV-2 S protein structure revealed by cryoEM (iv).

The above results suggest that the HR2 motif is included in the proteinase K-resistant stable core after the receptor-binding step (detected as a band at 46 kDa). Next, we predicted the position of the HR2 motif within the intermediate S protein. Previous studies show that receptor binding induces an “open” conformation in S1, which then affects the conformation of S2 (18, 19). The structure of the MHV-2 S protein at the pre-fusion or receptor-binding state was predicted from the cryoEM structure of the MHV-A59 S protein by homology modeling; furthermore, the amino acids in the S1 subunit that interact with the FPPR–HR1 region were estimated using “InterfaceResidues” in PyMol script. The FPPR–HR1 region in the S2 subunit detaches from the S1 subunit at amino acids V57, L59, T296, Q297, and N302, resulting in an “open” conformation of the S2 subunit after receptor binding (Fig. 5A), thereby enabling binding to the HR2 motif. The FPPR–HR1 regions of these two structures are cropped and shown as cartoons in Fig. 5B (i and ii).

Next, we predicted the structure of the FPPR-HR1 region that interacts with the HR2 motif in the intermediate S protein conformation. Artificial intelligence cannot be expected to predict a reliable protein structure for the unknown intermediate, but it can at least propose a candidate. The amino acid sequence of the MHV-2 S protein, from the N-terminus of the S1 subunit to the invariant motif of the S2 subunit, was connected to the HR2-peptide via a 35-glycine linker, and then the structure was predicted using Alphafold2 software (39). The S2 subunit was then cropped from the resulting structure (Fig. 5B, iii) and compared with the pre-fusion (Fig. 5B, i) and the receptor-binding states (Fig. 5B, ii). The FPPR–HR1 region complexed with the HR2 motif (i.e., the FPPR–HR1/HR2 complex) adopted a trimeric conformation (Fig. 5B, iii). The binding energy of the HR2 motif within the FPPR–HR1/HR2 complex (ΔiG = −13.7 kcal/mol) was weaker than that for the post-fusion 6HB complex (ΔiG = −29.9 kcal/mol), which was calculated from the PDBePISA website (40); this implies the FPPR-HR1/HR2 complex occurs temporarily during conformational changes and is ultimately replaced by a post-fusion structure. The FPPR–HR1/HR2 complex is presented here as a candidate for a transient metastable structure that forms within the S2 subunit after receptor binding.

In addition, we also examined the cleavage site that generates the 67 kDa band. A previous study shows that cleavage at K951 (postS2' site) of β48 (S_950–953_) within the FPPR during conformational changes produces the 67 kDa band (22). β48 forms a sheet with β47 (S_810–813_) in the pre-fusion state (Fig. 5B i), or a sheet with S_945-947_ in the post-fusion state (Fig. 5B, iv), making them uncleavable at the postS2′ site and resulting in the 69 kDa band only after cleavage at the S2′ site. During the conformational changes that occur after receptor binding, at least one β48 in the trimer needs to dissociate from β47; therefore, it is cleaved by trypsin at the postS2′ site and detected as the band at 67 kDa (Fig. 3B, upper panel, lane 1; and Fig. 4B, upper panel, lane 8). When the HR2-peptide is added after the receptor-binding step, the 67 kDa band is detected (Fig. 3B lane 16; and Fig. 4B upper panel lane 2), so it is presumed that β48 dissociates from β47 due to binding of the HR2-peptide, i.e., formation of the FPPR–HR1/HR2 complex. Both the 67 and 69 kDa bands were detected even in the presence of a saturating concentration of HR2-peptide (Fig. 3B, upper panel, lanes 15 and 16; and Fig. 4B, upper panel, lane 2), suggesting that two conformations of FPPR coexist after the receptor-binding step.

### Assembly of three HR1 motifs within the trimer at each step of the conformational change

**Fig 6:**
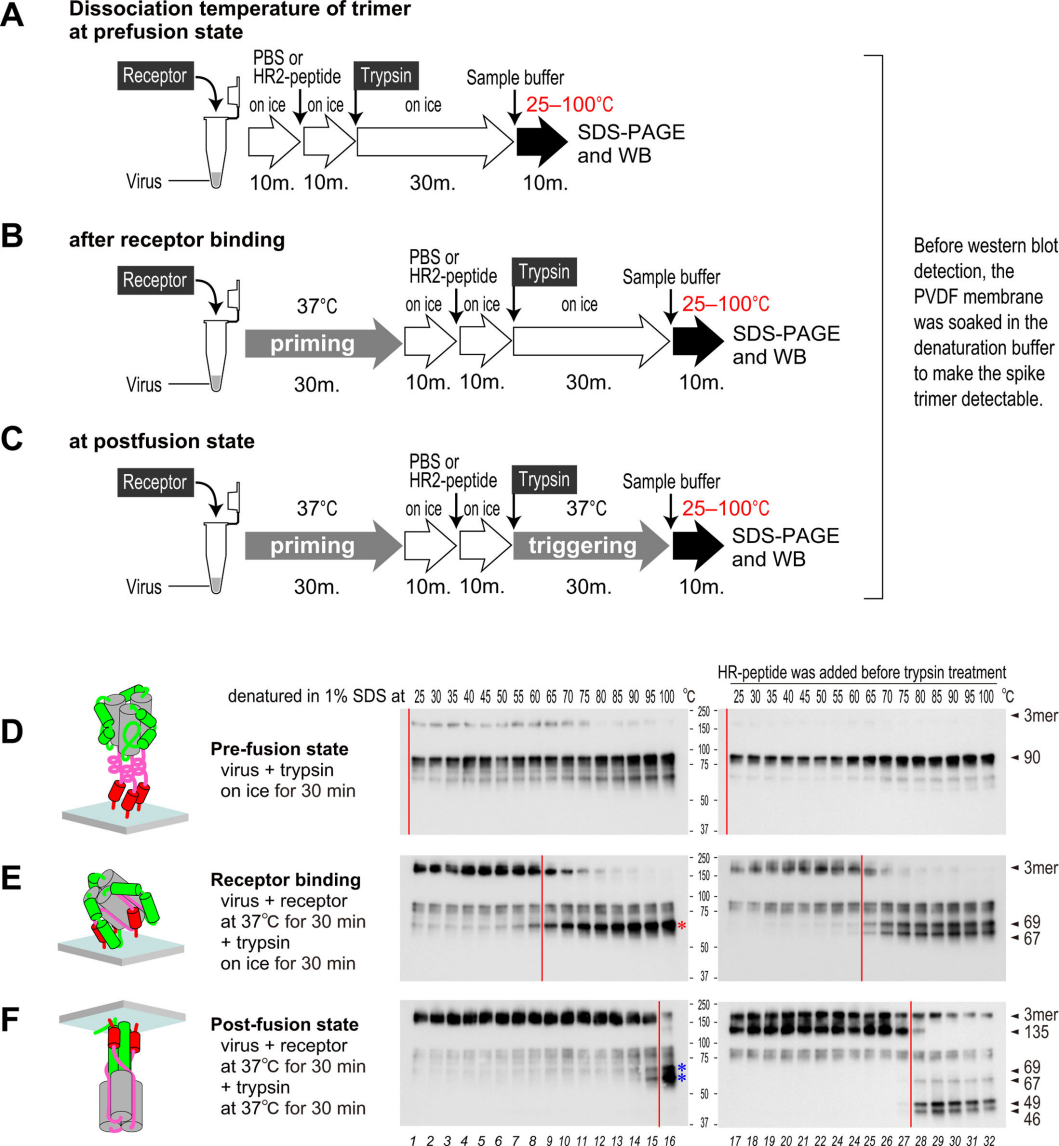
Thermostability of the MHV-2 spike trimer at each step of the conformational change. (A–C) Experimental flow chart illustrating detection of the thermostability of the trimer at each step of S protein activation (see panels D–F). (**D**) The pre-fusion state, (**E**) the receptor-binding state, and (**F**) the post-fusion state were generated in the presence (right panel) or absence (left panel) of the HR2-peptide. Reaction mixtures were treated with 5 mM Pefabloc SC and 1% SDS on ice and then incubated at the indicated temperatures for 10 min in a Veriti thermal cycler (Thermo Fisher). Western blot analysis was performed with the anti-CR antibody (MAb10G). The red bars indicate the temperature boundary at which half of the S protein dissociates to a monomer from a trimer.

The final form 6HB is a trimer comprising three tightly assembled HR1/HR2 motifs. To assess the strength of interaction within the trimer, the dissociation temperature of the S protein at each step of the conformational changes was examined by incubating the virus in 1% SDS. The experimental flow chart is shown in Fig. 6A through C. In the pre-fusion state, the trimer dissociated at <25°C (Fig. 6D, left panel). The dissociation temperature increased to 65°C following receptor binding, and then to 100°C following trypsin treatment (Fig. 6E and F, left panels), due to assembly of HR1 motifs within the trimer (as predicted by the deglycosylation experiment shown in Fig. 2). Of note, lane 13 of Fig. 6E and F shows that boiling with 1% SDS at 85°C distinguishes whether the HR1 motifs are assembled or not in the trimer; this property will be used to examine assembling of HR1/HR2 motifs (see the next paragraph). Interaction with the HR2-peptide did not affect the dissociation temperature of either the pre-fusion and receptor-binding states (Fig. 6D and E, right) although it did fall from 100°C to 80°C after trypsin treatment (Fig. 6F, lanes 16 and 28), suggesting that assembly of the HR1 motifs was blocked prematurely by the HR2-peptide. Otherwise, at the lower temperature, bands at 135 kDa appeared after trypsin treatment in the presence of the HR2-peptide (Fig. 6F, lanes 17–27). These bands are currently unexplained but may be caused by the formation of S protein dimers during the intermediate state.

### Packing and assembling of HR1/HR2 motifs

**Fig 7:**
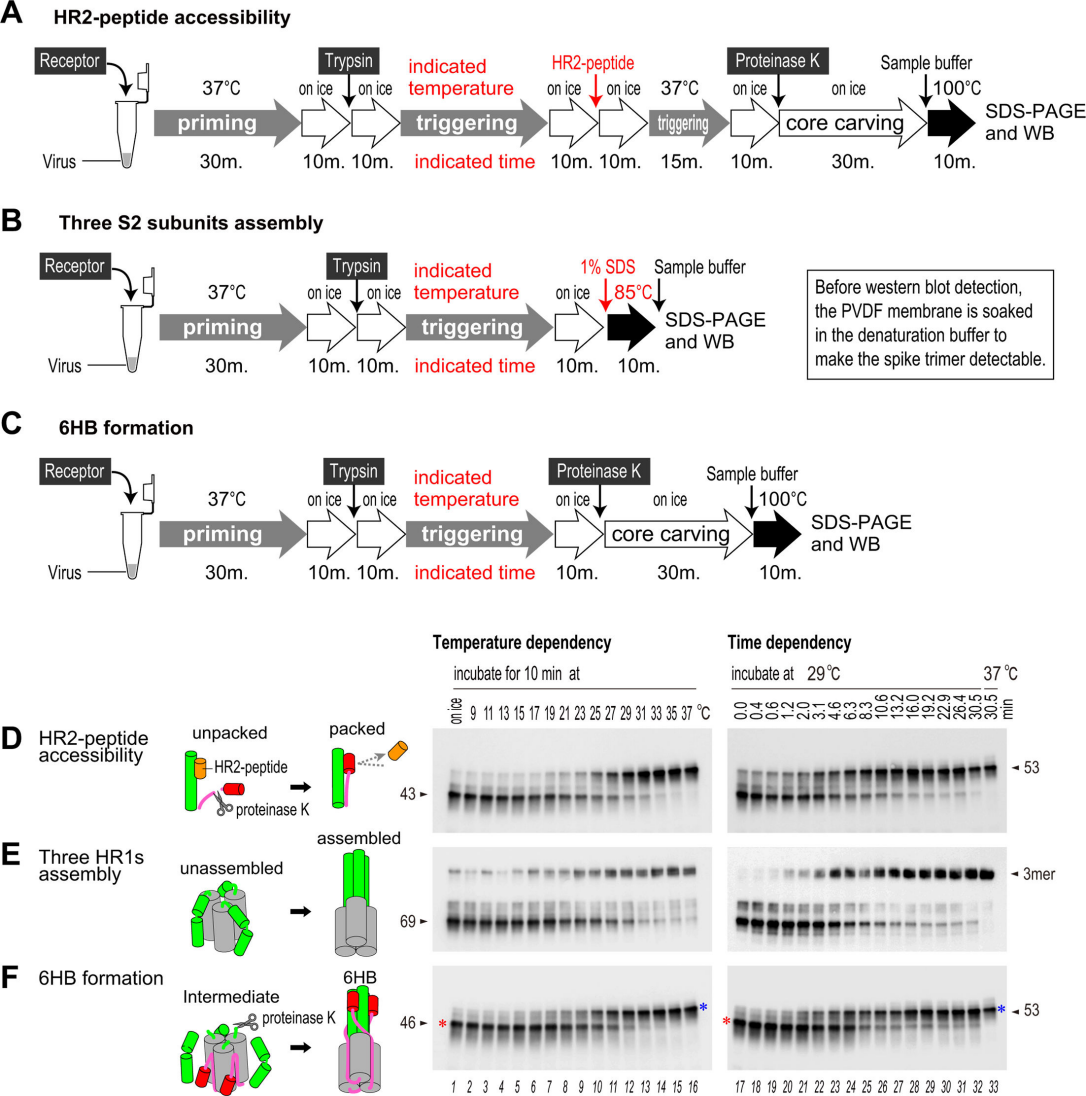
Packing and assembling of the HR1/HR2 motifs of the MHV-2 S protein. (A–C) Experimental flow chart illustrating the experiments used to detect packing and assembly of the HR1/HR2 motifs (see panels D–F). (**D**) HR2-peptide accessibility. The unpacked HR1/HR2 motif interacts with the HR2-peptide and produces the proteinase K-resistant band at 43 kDa, whereas the packed form produces the proteinase K-resistant band at 53 kDa. (**E**) Trimer assembly. The unassembled HR1 motif dissociates to form a 69 kDa monomer upon boiling at 85°C, whereas the assembled motif forms a trimer. (**F**) Formation of 6HB. The receptor-bound intermediate comprises a proteinase K-resistant 46 kDa fragment, whereas post-fusion 6HB comprises a 53 kDa fragment. To estimate the lowest temperature required to complete the conformational changes within 10 min, three reactions were conducted at various temperatures in a Veriti thermal cycler (left panels). Next, the time dependency of the conformational changes was tested at an optimum temperature of 29°C (right panels).

In light of the data presented above, we next examined conformational transitions of the S protein from the receptor-binding state to the post-fusion state, focusing on “each HR1/HR2 packing” and “three HR1s assembly”. For “each HR1/HR2 packing,” the interaction between the HR2-peptide and the “unpacked” HR1/HR2 motif generates the proteinase K-resistant 43 kDa protein (shown in Fig. 3B, lanes 8 and 16; and Fig. 4B, lanes 3 and 8), which is distinct from the 53 kDa protein representing the “packed” motif. For “three HR1s assembly,” boiling at 85°C in 1% SDS distinguished the “intermediate unassembled” S2 subunit from the “postfusion assembled” trimer (shown in Fig. 6E and F, lane 13, compare the middle and bottom panels). Additionally, core formation (whether “receptor-bound intermediate” or “post-fusion 6HB”) was detected as a 46 or a 53 kDa proteinase K-resistant band, respectively (shown in Fig. 1D lanes 15 and 16). The experimental flows are shown in Fig. 7A through C. To estimate the lowest temperature required for the conformational changes that lead to slow formation of 6HB, samples pretreated with the receptor (at 37°C) and trypsin (on ice) were incubated at various temperatures for 10 min (Fig. 7D through F, left panel). To assess time dependence, the samples were incubated at the lowest temperature, i.e., 29°C (Fig. 7D through F, right panel), and the reactions were paused at the indicated times by exposure to liquid nitrogen.

The results showed that three reactions, packing, assembling, and 6HB formation, occurred completely at temperatures higher than 31°C (Fig. 7D through F, left panels), but gradually and synchronously at 29°C, within 30.5 min (Fig. 7D through F, right panels). To enable more detailed evaluation under slower reaction conditions, the experiments were conducted at lower temperatures for 3 h (Fig. S3A through C). Packing and assembling were again gradual and synchronous. Remarkably, the specific temperature or time point at which the three HR1 motifs assemble without HR2 motif packing, the characteristic required to demonstrate formation of a homotrimeric pre-hairpin, was not identified (Fig. 7; Fig. S3). These observations suggest that “HR1/HR2 packing” and “three HR1s assembly” within the trimer occur almost simultaneously.

## DISCUSSION

Two results from the present study suggest that the three HR1 motifs do not assemble at the center to form a three-helical bundle after the receptor-binding step; first, the proteinase K-resistant 46 kDa fragment excludes the HR1 motif from the core (Fig. 2), and second, the interaction between the three S2 subunits in the trimer is weaker than that in the post-fusion 6HB (Fig. 6E and F). In addition, the cryoEM structure shows that the S2 subunit is covered by the S1 subunit during the receptor-binding step (protein data bank: 6VSJ), making it spatially impossible for three HR1 motifs to assemble. After treatment with trypsin on ice and then shifting the incubation temperature to 37°C, the HR1 motifs assemble at the center, thereby changing the proteinase K cleavage site from the C-terminal side to the N-terminal side of the HR1 motif, allowing strong interaction between the three S2 subunits, thereby generating the proteinase K-resistant 53 kDa band representing 6HB. Otherwise, we did not obtain data that allow us to predict the structure of the connector region although it forms a core comprising the proteinase K-resistant 46 kDa protein after the receptor-binding step (Fig. 2). We assume that the leash of the connector region is packed into the groove of the invariant motif during the receptor-binding step, as observed in the post-fusion structure (protein data bank: 6B3O).

**Fig 8:**
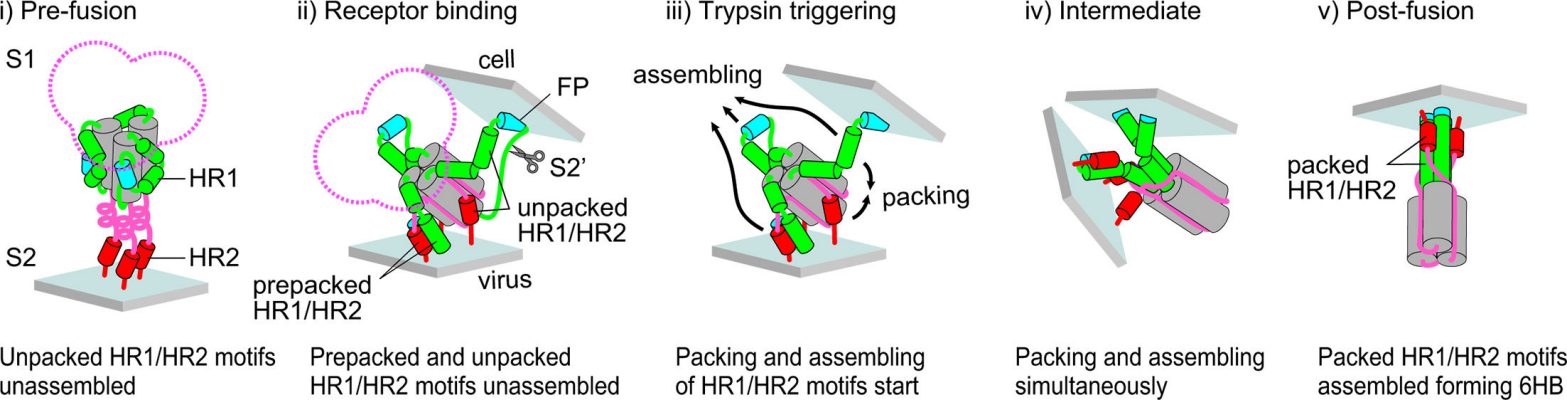
Model of conformational changes. The pre-fusion state of the S protein is a symmetric trimer (**i**), which changes to an asymmetric trimer during the receptor-binding step (the receptor-bound trimer comprises packed and unpacked HR1/HR2 motifs) (ii). Trypsin treatment dissociates the S1 subunit and triggers subsequent conformational changes (iii). Then, packing of the remaining unpacked HR1/HR2 motifs, followed by assembly of the three HR1/HR2 motifs, occurs simultaneously (iv), followed by direct formation of the 6HB structure (**v**). FP, fusion peptide; HR1/HR2, heptad repeats.

In a previous study, we demonstrated that the 67 and 69 kDa products exhibit distinct conformations. The 69 kDa undergoes degradation in response to binding to the HR2-peptide, suggesting that it is likely to possess an unpacked HR1/HR2 motif (22). By contrast, the 67 kDa fragment showed lower susceptibility to the HR2-peptide, leading to possession of a packed HR1/HR2 motif (22). Here, we also demonstrated that even in the presence of saturating concentrations of the HR2-peptide, half of the S2 subunit was detected as a 67 kDa band and half as a 69 kDa band (Fig. 3B, lane 16; and Fig. 4B, lane 2). This suggests the coexistence of prepacked and unpacked HR1/HR2 motifs after the receptor-binding step. Furthermore, the two-dimensional SDS-PAGE experiment conducted in our previous study revealed coexistence of the 69 and 67 kDa proteins within the trimer (22). The pattern of these two bands (67 kDa and 69 kDa) was the same when HR1 bound to the endogenous HR2 motif instead of the exogenous HR2-peptide, i.e., when the spike protein forms 6HB after undergoing conformational changes (Fig. 3B lane 1, and Fig. 4B lane 8). Therefore, the coexistence of prepacked and unpacked HR1/HR2 motifs, along with their unassembled HR1 motifs, within a trimer occurs during the receptor-binding step (Fig. 8ii). The structure of the prepacked HR1/HR2 motif in the S2 subunit was predicted using AlphaFold2 software (Fig. 5Biii), implying that the HR1/HR2 motif can form a transient complex with the FPPR region (i.e., the FPPR–HR1/HR2 complex) at the groove that appears after receptor binding (Fig. 5A). Of note, our model was predicted based on experimental data obtained from the uncleaved S protein of MHV-2. The flexibility of the S2 conformation after receptor binding may differ between the uncleaved and cleaved forms, potentially leading to distinct conformational changes.

The typical homotrimeric pre-hairpin model for the class I fusion protein was predicted based on trimerization of synthesized HR1 and HR2-peptides (30, 41); however, this has never been observed using authentic viruses. CryoEM tomography has captured the intermediate state of the retrovirus Env, paramyxovirus F, and influenza virus HA proteins, but the images obtained are not clear enough to confirm a homotrimeric pre-hairpin structure (42–48). A recent study using cryoEM tomography captured the intermediate state of the SARS-CoV-2 S protein by halting refolding of HR1/HR2 using a membrane-anchored HR2-peptide; the data seem to suggest that the S protein forms a homotrimeric pre-hairpin structure (29). However, we found that in the presence of the HR2-peptide, the S protein formed a proteinase K-resistant core detected at 53 kDa after the receptor-binding step (Fig. 3B, lane 16), and (at least partially) formed a stable trimer after incubation with trypsin at 37°C (Fig. 6F, right), which dissociated at 80°C, suggesting that the HR2-peptide affects the conformation of the S protein. Therefore, the S protein detected in the presence of HR2-peptides cannot be claimed conclusively to be an intermediate form because the HR2-peptide itself affects the conformation of the S protein.

If the S protein forms the typical homotrimeric pre-hairpin intermediate, then the HR2 region must be highly flexible to enable conformational changes without steric hindrance; however, our results indicate that the HR2 region adopts a proteinase K-resistant stable conformation after the receptor-binding step (detected as the 46 kDa band). Furthermore, the results in Fig. 7 show synchronized timing of the loss of HR2-peptide accessibility, along with stable trimer formation, after trypsin treatment. This suggests that packing of the HR1/HR2 motif and assembly of the HR1 motifs occur almost simultaneously. If the typical homotrimeric pre-hairpin is formed, “three HR1s assembly” must occur earlier than “HR1/HR2 packing,” but we did not observe this. In our model, the trimer formed after the receptor-binding step contains prepacked and unpacked HR1/HR2 motifs, in which HR1s are not assembled at a center and at least one FP is inserted into the target membrane. It is likely, therefore, that the asymmetric-unassembled S2 trimer arises during the receptor-binding step, thereby breaking the rotational symmetry of the homotrimer and enabling compact transition of the S2 subunit from the pre-fusion to the post-fusion conformation (Fig. 8ii). Then, proteases such as trypsin trigger release of the “loaded springs” to facilitate direct assembly of the HR1/HR2 motifs (Fig. 8iii), followed by the formation of 6HB (Fig. 8v). Taken together, the data presented herein suggest an alternative to the “homotrimeric pre-hairpin” model for the S protein intermediate. Our model, which indirectly infers existence of an intermediate conformation of the S protein based on western blotting, will need to be verified by direct detection using advanced structural biology techniques.

## MATERIALS AND METHODS

### Viruses, cells, soluble receptors, HR2-peptides, and antibodies

The SARS-CoV-2 ancestral strain (hCoV-19/Japan/TY-WK-521/2020) was propagated in VERO-E6/TMPRSS2 cells cultured in Dulbecco’s modified Eagle’s medium (DMEM, D5796; Sigma-Aldrich). Viruses were collected at 21 h post-infection and stored at –80°C until required for experiments. The recombinant soluble SARS-CoV-2 receptor (human ACE2, amino acids (aa) 1–740) was cloned into the pCAGGS/MCS vector (49) and then transfected into Expi293F cells using the ExpiFectamine 293 transfection kit (ThermoFisher). The culture supernatant was harvested and applied to a HiTrap Q HP column (Cytiva) coupled to an AKTA FPLC apparatus (Cytiva) and concentrated using a Vivaspin 20–30K (Sartorius). The HR2-peptide (DISGINASVVNIQKEIDRLNEVAKNLNESLIDLQEL) was synthesized (GenScript) and dissolved in water (36). An antibody specific for the SARS-CoV S2 subunit (aa 1029–1192) was purchased from GeneTex (GTX632604).

MHV-2 was propagated in DBT cells cultured in DMEM (05919; Nissui) containing 10% tryptose phosphate broth (BD Difco) (22). Viruses were collected at 21 h post-infection and stored at –80°C. The soluble form of the MHV receptor (CEACAM1a) was produced using recombinant baculovirus and purified as described previously (50). The HR2-peptide DLSLDFEKLNVTLLDLTYEMNRIQDAIKKLNESYINLKE was provided by BJ Bosch (Utrecht University) (35). A biotinylated-HR2-peptide was prepared using EZ-Link Sulfo-NHS-LC-Biotin (21335; Pierce). Three anti-MHV S protein antibodies were used: a mouse monoclonal antibody recognizing the 10G epitope [MAb-10G; provided by SG Siddell (Institute of Virology, Würzburg)] (51), a rabbit anti-HR2 antibody (named EC3; derived from a rabbit immunized with a 19 aa peptide DLTDEMNRIQDAIKKLNES; Eurofins), and a rabbit anti-CT antibody (obtained from a rabbit immunized with the 11 aa peptide IVIHNISSHED; Eurofins).

### Induction of conformational changes in the S protein

#### Standard reaction

To induce conformational changes in the S protein, a 10 µL solution of authentic MHV-2 or SARS-CoV-2 (10^6^ PFU/10 µL) was mixed with 1.1 µL of soluble receptor (10 µM) and incubated at 37°C for 30 min. To induce conformational changes, 1.2 µL of trypsin (100 µg/mL, T8802; Sigma) was added, followed by incubation at 37°C for 30 min. To evaluate the proteinase K-resistant core of the S protein, the reaction mixtures were chilled on ice for 10 min, followed by the addition of 1.4 µL of proteinase K (1 mg/mL, 166-21051; Wako) and incubation for 30 min on ice. Next, 3.4 µL of sample buffer comprising 30% glycerol, 250 mM Tris (pH 6.8), 2.5% SDS, a small amount of bromophenol blue, 100 mM DTT, and 1 mM Pefabloc SC (11429868001; Roche) was added to the reaction, and the mixture was boiled at 100°C for 10 min. Samples were applied to an SDS-PAGE gel (10% e-PAGEL; ATTO), transferred to a PVDF membrane (Immun-Blot; Bio-Rad), and soaked for 30 min in ImmunoBlock (CTKN001; DS Pharma Biomedical). Western blot analysis was carried out using anti-S2 antibodies, followed by horseradish peroxidase-conjugated anti-mouse (32430; ThermoFisher Scientific) or anti-rabbit (sc-2054; Santa Cruz Biotech) IgG. Immunoreactive bands were visualized with a chemiluminescence kit (34095; ThermoFisher Scientific) and a LAS-3000 instrument (Fuji film, Japan). Flowcharts showing the respective experiments are shown in Fig. S1.

#### Protein denaturation on unboiled samples on PVDF membranes

After SDS-PAGE of unboiled samples and electrotransfer to a PVDF membrane, the membrane was soaked in stripping buffer (46430; ThermoFisher Scientific) at room temperature for 10 min to denature the S protein, rinsed 10 times with phosphate-buffered saline (PBS; BR0014G; Oxoid), and blocked with ImmunoBlock solution.

### Deglycosylation of the S2 subunit

Virus samples treated with receptor, trypsin, and proteinase K were prepared as described above, and deglycosylation was carried out using protein deglycosylation mix II (P6044S; NEB, UK).

### Time of HR2-peptide addition to block the MHV-2 cell entry

DBT cells (10^6^ cells) were detached from the culture bottle using nonenzymatic cell dissociation solution (C5914; Sigma), pelleted by centrifugation, mixed with 10^6^ PFU MHV-2, and then left on ice for 30 min. The cells were pelleted again by centrifugation and resuspended in PBS containing 40 µM E64d. An 8 µL solution containing 10^4^ cells was placed in an 8-well tube (673210; Greiner) on an ice-cold metal rack and left for 5 min. To induce the first conformational change in the viral S protein, the cells were incubated at 37°C for 5 min using a Veriti thermal cycler (Thermo). Next, 2 µL of trypsin (50 µg/mL; final concentration, 10 µg/mL) was added, and the mixture was left on ice for 10 min. To induce the second conformational change in the S protein, the cells were shifted to 37°C and incubated for 30 min. At the time points indicated in the figure, 2.5 µL of 100 µM HR2-peptide was added (final concentration, 20 µM). The cells were then resuspended in 100 µL DMEM containing 40 µM E64d, 40 µM camostat, and 5% fetal bovine serum, seeded in collagen-coated 96-well plates (4860-010; Iwaki), and incubated at 37°C for 5 h. Cellular RNA was isolated from a well of the 96 well plate using Isogen (311-02501; Nippon Gene). Real-time reverse transcription-PCR was performed as described previously to estimate the amount of mRNA encoding the MHV-2 N gene (10).

### Binding of the biotinylated-HR2-peptide to MHV-2

An 18 µL of MHV-2 was mixed with 2 µL of soluble receptor (10 µM) and 2.2 µL of trypsin (100 µg/mL) to induce conformational changes. The reaction mixtures from each step of conformational changes were then treated with 1.2 µL of Pefabloc SC (1 mM) on ice for 10 min, followed by 2.6 µL of biotinylated-HR2-peptide (10 µM) on ice for 10 min, and 6.5 µL of streptavidin-magnetic beads (M-1003-010; Vector) on ice for 10 min. After washing three times with PBS (pH 7.4), viral RNA was isolated from the reaction mixture using Isogen (311-02501; Nippon Gene). Real-time reverse transcription-PCR was performed as described previously to estimate the amount of MHV-2 N gene (10).

### Prediction of the S protein structure

Residues at the interface between the FPPR–HR1 region and the S protein globule were analyzed using PyMol script “InterfaceResidues,” https://pymolwiki.org/index.php/InterfaceResidues. The cryoEM structure of the MHV-A59 S protein during the receptor-binding step (protein data bank: 6VSJ) was compared with that in the prefusion state (protein data bank: 3JCL). To predict the intermediate structure of the FPPR–HR1/HR2 in the S2 subunit, the amino acid sequence of the MHV-2 S protein (GenBank: U72635.1) from the N terminus of the S1 subunit to the invariant motif of the S2 subunit (aa 1–1154) was connected to the HR2 motif DLSLDFEKLNVTLLDLTYEMNRIQDAIKKLNESYINLKE (aa 1151–1285) via a 35 glycine linker. Next, the simulation was carried out using AlphaFold2 with the ColabFold v1.5.2-patch. The binding energy of the HR2 in the complex was calculated using the PDBePISA website: https://www.ebi.ac.uk/pdbe/pisa/. To construct the MHV-2 S protein model, the cryo-electron structure of the MHV-A59 S protein or the SARS-CoV-2 S protein indicated in Fig. 4 was modified by homology modeling (52) using the MOE software package (Chemical Computing Group, Montreal, Canada). Structural figures were then generated using the PyMOL molecular visualization system.

### Statistical analysis

Statistical significance was assessed using a two-tailed Student’s *t*-test. A *P*-value of <0.05 was considered significant (n.s., not significant; *, significant [*P* ≤ 0.05]; **, highly significant [*P* ≤ 0.01]). Error bars indicate the standard deviations.

## SUPPLEMENTAL MATERIAL

Supplemental figures mbio.01203-24-s0001.docx Fig. S1-S3.

ASM does not own the copyrights to Supplemental Material that may be linked to, or accessed through, an article. The authors have granted ASM a non-exclusive, world-wide license to publish the Supplemental Material files. Please contact the corresponding author directly for reuse.
