# Supplementary material for: Biochemical analysis of packing and assembling heptad repeat motifs in the coronavirus spike protein trimer
Source: mBio. 2024 Oct 23;15(11):e01203-24. doi: 10.1128/mbio.01203-24 (PMC11559096; doi:10.1128/mbio.01203-24)
Supplement: Supplemental figures — Fig. S1-S3. [file mbio.01203-24-s0001.docx]

**Supporting Information for**

**Biochemical Analysis of Packing and Assembling Heptad Repeat Motifs in the Coronavirus Spike Protein Trimer**

Jun Kobayashi, Kazuhiko Kanou, Hiyori Okura, Tahmina MST Akter, Shuetsu Fukushi, and Shutoku Matsuyama

This file includes:

Figures S1 to S3


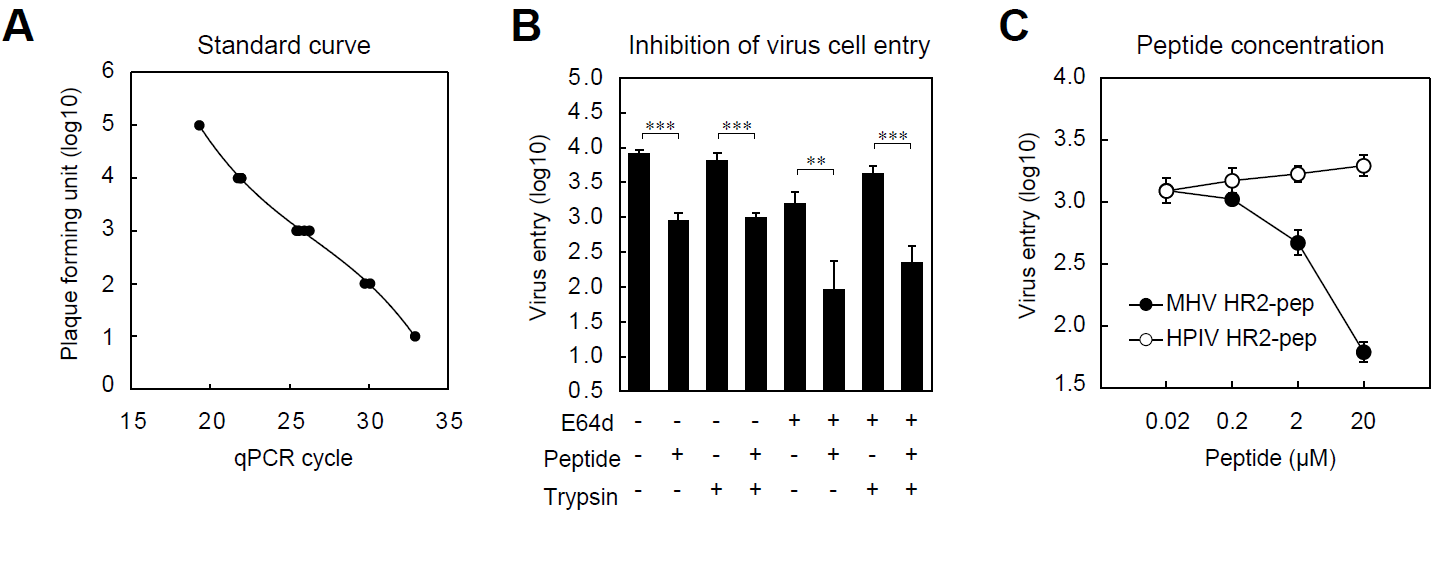


**Fig. S1. Interaction between the HR2-peptide and the MHV-2 S protein.**

**(A)** Standard curve for the virus cell entry assay. DBT cells were infected with MHV-2 (serially diluted 10-fold), cellular RNA was collected after 5 h, and viral RNA was quantified by real-time PCR. The relationship between the number of viral infectious units and the amount of RNA is indicated in the graph. **(B)** Inhibition of virus cell entry. MHV-2 was adsorbed onto DBT cells pretreated with E64d or a vehicle control, and then cultured for 5 h in the presence or absence of 5 µg/mL trypsin after addition (or not) of 20 µM HR2-peptide. Cellular RNA was extracted, and viral RNA was quantified by real-time PCR. **(C)** Virus cell entry depends on the HR2-peptide concentration. The virus cell entry assay was performed as described above in the presence of 40 µM E64d and the HR2-peptide (serially diluted 10-fold). The HR2-peptide of human parainfluenza virus (HPIV) was used for comparison.

**Fig. S2. Interaction between the HR2-peptide and the SARS-CoV-2 S protein.**

**(A)** Standard curve for the virus cell entry assay. VeroE6 cells were infected with diluted SARS-CoV-2 (serially diluted 10-fold). Cellular RNA was collected after 5 h and viral RNA was quantified using qPCR. The relationship between the number of viral infectious units and the amount of RNA is shown in the graph. **(B)** Inhibition of virus cell entry. SARS-CoV-2 was adsorbed to VeroE6 cells pretreated with E64d or a vehicle control, and then cultured for 5 h in the presence or absence of 5 µg/mL trypsin after addition (or not) of 200 µM HR2-peptide. Next, cellular RNA was collected, and viral RNA was quantified using qPCR. **(C)** HR2-peptide concentration dependency. A virus cell entry assay was performed in the presence of 40 µM E64d using the method described above with HR2-peptide serially diluted 10-fold. The HR2-peptide of human parainfluenza virus (HPIV) was used for comparison. **(D)** An experimental flow chart illustrating treatment with the HR-2 peptide treatment. **(E)** Effect of the HR2-peptide on the conformation of the S2 subunit. During experimental activation of the S protein, the HR2-peptide (serially diluted 5-fold) was added after the receptor binding step, followed by trypsin treatment. Next, samples were treated with proteinase K to carve out the core structure. The S2 subunit was detected by western blot analysis using a specific antibody (GTX632604).

**Fig. S3. Packing and assembling the HR1/HR2 motifs within the MHV-2 S protein at low temperature.**

**(A)** HR2-peptide accessibility. The unpacked HR1/HR2 motif interacts with the HR2-peptide and produces the proteinase K-resistant band at 43 kDa, whereas the packed form produces the proteinase K-resistant band at 53 kDa. **(B)** Trimer assembling. Upon boiling at 85°C, the unassembled HR1 motif dissociates to form a 69 kDa monomer, whereas the assembled motif forms a trimer. **(C)** Formation of 6HB. In addition, the receptor-bound intermediate comprises a proteinase K-resistant 46 kDa fragment, whereas post-fusion 6HB comprises a 53 kDa fragment. To estimate the lowest temperature required for conformational changes in the MHV-2 S protein within 3 h, three reactions were conducted at various temperatures controlled by a Veriti thermal cycler (left panels). Next, the time dependency of the conformational changes was tested at an optimum temperature of 24°C (right panels).
